# PvTFDB: a *Phaseolus vulgaris* transcription factors database for expediting functional genomics in legumes

**DOI:** 10.1093/database/baw114

**Published:** 2016-07-27

**Authors:** Bhawna, V.S. Bonthala, MNV Prasad Gajula

**Affiliations:** Institute of Biotechnology, PJTSAU, Rajendra Nagar, Hyderabad 500030, India

## Abstract

The common bean [*Phaseolus vulgaris* (L.)] is one of the essential proteinaceous vegetables grown in developing countries. However, its production is challenged by low yields caused by numerous biotic and abiotic stress conditions. Regulatory transcription factors (TFs) symbolize a key component of the genome and are the most significant targets for producing stress tolerant crop and hence functional genomic studies of these TFs are important. Therefore, here we have constructed a web-accessible TFs database for *P. vulgaris*, called PvTFDB, which contains 2370 putative TF gene models in 49 TF families. This database provides a comprehensive information for each of the identified TF that includes sequence data, functional annotation, SSRs with their primer sets, protein physical properties, chromosomal location, phylogeny, tissue-specific gene expression data, orthologues, *cis*-regulatory elements and gene ontology (GO) assignment. Altogether, this information would be used in expediting the functional genomic studies of a specific TF(s) of interest. The objectives of this database are to understand functional genomics study of common bean TFs and recognize the regulatory mechanisms underlying various stress responses to ease breeding strategy for variety production through a couple of search interfaces including gene ID, functional annotation and browsing interfaces including by family and by chromosome. This database will also serve as a promising central repository for researchers as well as breeders who are working towards crop improvement of legume crops. In addition, this database provide the user unrestricted public access and the user can download entire data present in the database freely.

**Database URL:** http://www.multiomics.in/PvTFDB/

## Introduction

The common bean [*Phaseolus vulgaris* (L.)] is a self-pollinated species belonging to the *Fabaceae* family with a small genome size of 588 MB and diploid genotype (2*n* =2*x* =22) (1). It is one of the most important ancient legumes cultivated species of the genus *Phaseolus* and is highly consumed in various developing countries due to its nutritional components. It is known for its high nitrogen fixation ability from the atmosphere with the help of symbiotic nitrogen-fixing bacteria. Nitrogen fixation ability reduces the need of synthetic crop fertilizers hence reduce water pollution caused by leaching. Moreover, the common bean is a vital component of food security plans directing to deliver better human nutrition in developing countries (2). In addition, this plant is useful not only for the consumption of humans for its nutritional value but also as a source of therapeutic and traditional medicine. Therefore, cultivation of *P.* *vulgaris* (L.) is beneficial in both economic, health and environmental perspective. However, low harvests, sudden climate change and various stress factors distressing the production and not meeting the huge demand for the crop. As the demand to produce stress tolerant varieties of the crop is escalating, we still lack the complete knowledge on regulatory mechanism to develope a transgenic crop. However, it is known that TFs are the master regulators for various molecular mechanisms involved in both abiotic and biotic stresses in plants. Therefore, it is highly necessary to attain proper knowledge of various TFs as their indirect manipulation is helpful in improving local breeding programmes. TFs and related *cis*-regulatory sequences are controlling factors for gene expression and regulation for their temporal and spatial expression during various biological processes comprising both abiotic and biotic stress factors (3, 4). Because of their importance in regulation and controlling of various molecular mechanisms, it is necessary to categorize TFs and study all the common bean genes involved in transcriptional control. In this study, we categorized and developed a comprehensive TF database called *P.* *vulgaris* Transcription Factors Database (PvTFDB) by using various computational methods. We utilized the recently published common bean's genome sequence data (5) to develope PvTFDB. This database can serve as a resource in utilizing functional genomic information of TFs in genetic engineering and breeding for the developement of stress tolerant plants varieties.

PvTFDB is the first online comprehensive TF database developed for the legume crop, common bean. This database contains a set of 2370 identified TFs (Gene models) and classifies them into 49 TF families. Promisingly, this PvTFDB will assist in molecular examination of transcriptional mechanisms in common bean, specifically in identification and characterization of TFs regulating the expression of various genes. TFs also considerate for the signal transduction events and ultimately leading towards development of new crop varieties by involving genetic manipulation. However, a complete study on common bean TF(s) for stress responses is not available until now and that information is a prerequisite for detailed molecular level investigations. PvTFDB will serve as a central resource to all the scientists and breeders who are working towards improving legumes crops.

## Methods and database contents

### Identification of putative TFs

The common bean’s whole proteome data containing about 31 638 predicted proteins along with their CDS, primary transcript sequences. The whole genome sequences were downloaded from Phytozome v11 ( https://phytozome.jgi.doe.gov/pz/portal.html). The complete set of TF sequences present in Plant Transcription Factor Database (http://plntfdb.bio.uni-potsdam.de/v3.0) were retrieved and HMM profiles were created for each of the TF family using HMMER suite (6). The HMM profile was then searched against the common bean’s whole proteome data using HMMER suite with default *E*-value (6). The raw alignment data is examined manually to ensure reliability. A total of 2370 putative TFs were extracted and are characterized into 49 families. The complete sequence data of each TF family including genomic DNA, CDS, primary transcript, amino acid and 2 kb of 5′-upstream sequence is made available to users and can be downloaded from PvTFDB.

### Annotation of TFs and tissue-specific expression

We performed annotation studies at gene, protein and family levels to develop comprehensive information for the identified putative TFs. The individual TF gene sequences were examined by local nucleotide BLAST search (7) against the downloaded whole genome sequences of common bean; and to acquire corresponding physical positions on individual chromosomes. The individual TF protein sequences were scanned using standalone version of InterProScan (8), that contains various functional databases including Pfam (http://pfam.xfam.org/), PRINTS (https://www.ebi.ac.uk/interpro/), SMART (http://smart.embl-heidelberg.de/), PrositeProfiles (http://pir.georgetown.edu/), InterProScan (https://www.ebi.ac.uk/interpro/), Phytozome (http://phytozome.net/), Panther (http://www.pantherdb.org/panther/), Gene3D (https://www.ebi.ac.uk/interpro/signature/), gene ontology (GO) (http://amigo.geneontology.org/) and SUPERFAMILY (http://supfam.cs.bris.ac.uk/SUPERFAMILY/), to predict the functional domains, motifs along with their structural annotation and permits the user to develop comprehensive information related to a respective TF. GOs were assigned to each of the putative TF using Blast2GO (9). To identify the presence of different *cis*-regulatory elements in the promoter region, a 2 kb upstream region of each common bean TF gene was examined using PLACE database (http://www.dna.affrc.go.jp/PLACE/index.html). Based on the existing information in the literature, the recognized *cis*-regulatory elements were characterized according to gene expression responsive elements and other motifs in PvTFDB. A variety of gene regulatory elements exist in the promoters region of different TF(s) were identified. The combination of *cis*-regulatory elements with gene expression data provides insight into the regulatory interactions among TFs; and other various proteins regulating gene expression in common bean. The normalized RPKM (RNA-Seq based) information of seven tissues of common bean (root, nodule, leaf, stem, pod, seed and flower) were retrieved using Common Bean Gene Expression Atlas (http://plantgrn.noble.org/PvGEA/) (10) and was used to produce heatmaps and graphs for gene expression in PvTFDB at TF family and at individual TF gene level, respectively.

### Phylogeny, SSRs and orthologues

For phylogenetic analysis, amino acid sequences of each TF family were uploaded into MEGA5. ClustalW was used to perform multiple sequence alignments with default parameters. Then the aligned amino acid sequences file was used to draw a unrooted phylogenetic tree by using bootstrap examination for 1000 replicates on neighbour-joining method (11–13). The phylogenetic tree of corresponding TF family members can be envisaged in PvTFDB. The CDS sequences of putative TFs were scanned for SSRs (Simple Sequence Repeats) using MISA tool (http://pgrc.ipk-gatersleben.de/misa/). The identified SSRs along with their flanking regions (150 bp) were used to design primers using Primer3 tool (http://simgene.com/Primer3). In order to find out the orthologues to each of the identified putative TFs, the protein sequences were searched with protein BLAST (7) against various legume crops including soybean, pigeon pea, *Lotus japonicus*, adzuki bean and mung bean with default parameters. Homology of >80% were considered as a significant threshold to consider as orthologue.

### Database construction and implementation

**Figure 1.**
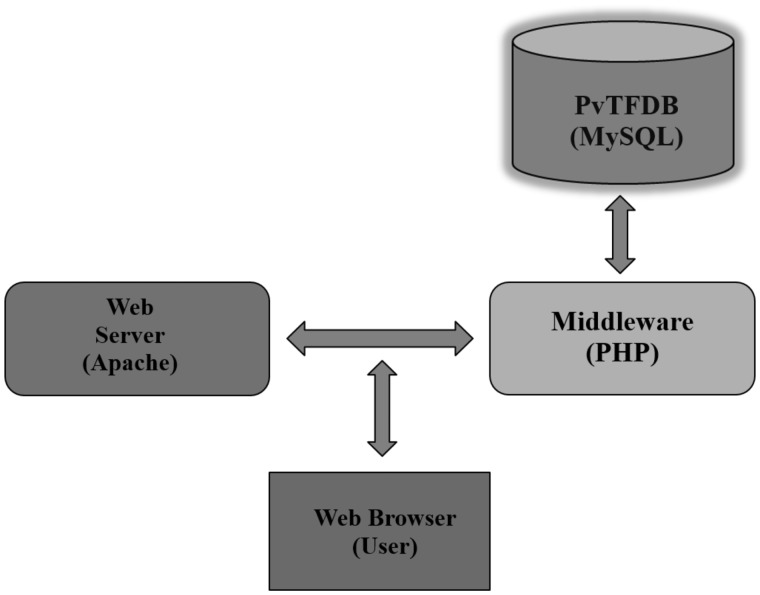
The three-tier architecture of PvTFDB: in this database, open-source programmes (PHP/MySQL) were used.

**Figure 2.**
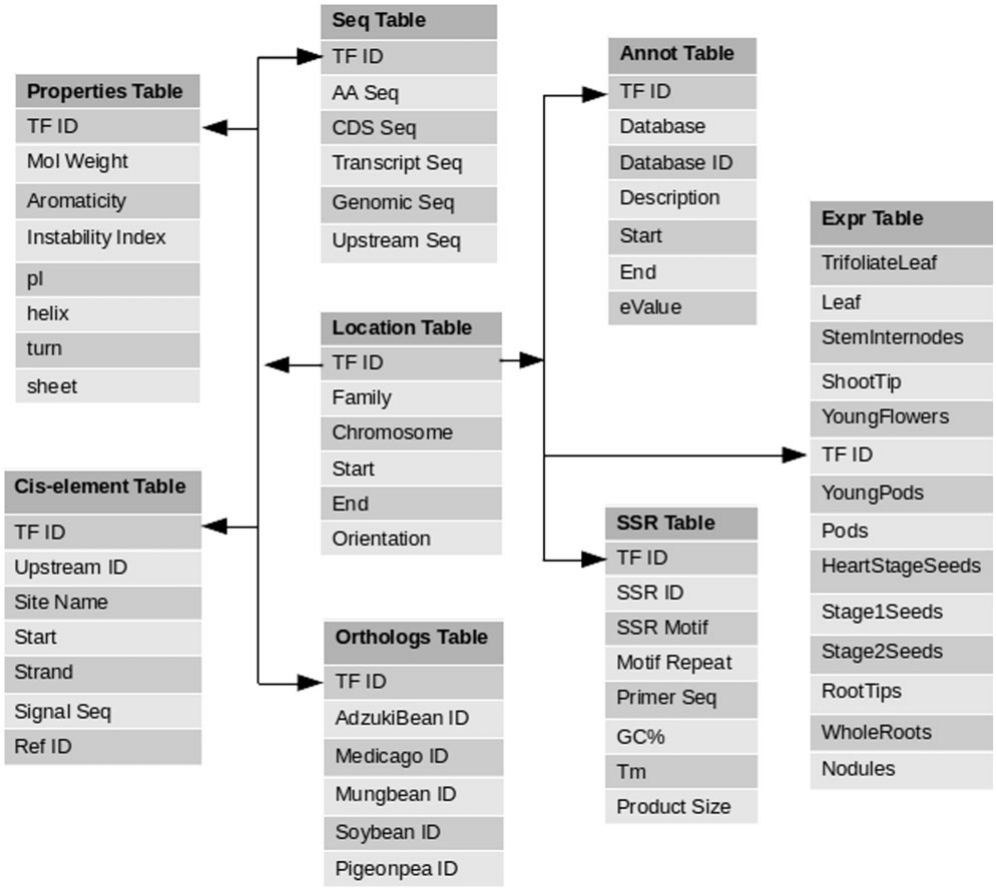
Database schema diagram of PvTFDB: this schema digram contains a total of eight tables which contains comprehensive information about each TF deposited in PvTFDB.

The PvTFDB web server was set up by the three-tier architecture concept using Apache/PHP/MySQL on a Linux platform (Figure 1). To ease the utility of database, all the data/results were deposited in MySQL relational database for custom search and retrieval of data. A pictorial demonstration of data incorporated (database schema) in the PvTFDB is shown in Figure 2. The graphs used to visualize gene expression was produced using PHP’s graph library called pChart (http://www.pchart.net/).

## Results and discussion

The complete TFs data can be browsed by chromosomes or families level. TFs search can be done by using gene ID or gene function ID. A short introduction of each TF family with a hyperlink to respective literature is available for users to briefly understand each TF family. In addition, a custom search feature for browsing the PvTFDB using GO ID, Pfam ID, SMART ID, InterProScan ID or ProSiteProfiles ID is also provided. A tutorial has also been provided under ‘Tutorial’ tab to increase the usability of the database (Supplementary Materials). The database can be accessed freely by using any standard web browsers.

### Search by using gene ID and function ID

**Figure 3.**
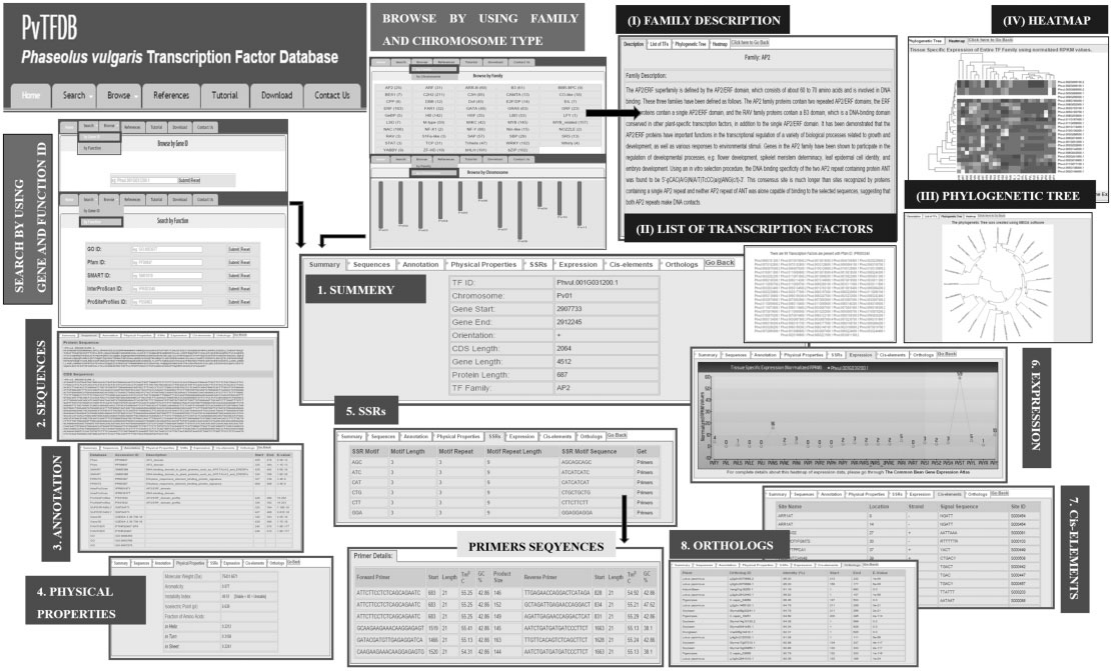
Pictorial representation of search and browse options with results of PvTFDB: the figure presents how the database can be searched/browsed to find out the details of each TF deposited in PvTFDB and are (i) Search by gene ID or function, (ii) Browse by TF family or by Chromosome. All these options gives comprehensive information about each TF. For complete description of each option, please go through the ‘Tutorial’ tab in the database.

The gene ID search selection facilitates the user to search the TFs based on gene ID of interest (Figure 3). After submitting gene ID, the database will present the complete details of TF. Similarly by using function ID, the user can search PvTFDB by entering GO ID, Pfam ID, SMART ID, InterProScan ID or ProSiteProfiles ID of interest (Figure 3). After submitting the function ID, a page will open presenting a list of TFs details which contain the specific function. Further clicking on a particular TF ID, a web page will direct the user to complete data details to ‘TF Details Page’ linked to that particular TF ID. This page contains comprehensive information that includes a summary of TF including chromosomal location, sequences, annotation, physical properties, SSRs, expression, *cis*-elements and orthologues in ‘tab’ format.

### Browse by TF family-wise and chromosome-wise

This browsing option eases the user to examine the TFs based on their family or chromosome (Figure 3). All the 2370 TFs are categorized into 49 families. The ‘TF Family Details’ page begins with the family name, the family description, the hyperlink to published literature, the list of TF(s) present in TF family of interest, phylogenetic tree and heatmap diagram representing gene expression levels across different tissues for all the TF members present in a specific TF family. Further, a few more options are available to understand the various details of TFs like sequences, annotation, physical properties, SSRs, expression, *cis*-elements and orthologues. Similarly, the TFs were categorized according to their chromosomal location on the common bean genome and this allows the user to search the TFs by clicking on ‘chromosome’ of interest (Figure 3). It will enlist the entire number of TFs present in the given chromosome with its ID, orientation, family name, CDS, gene, protein length, including start and end position on a chromosome. These details will provide the complete information of the given TF including its basic data of chromosomal location, protein information, protein functional annotation, GO annotation, tissue-specific gene expression data and complete sequence information.

Considering the importance of common bean as a highly demanding nutritional crop, it is a prerequisite to study the functional genomics of this legume crop. Two independent groups namely the US Department of Energy-Joint Genome Initiative and the US Department of Agriculture had sequenced its genome and transcriptome, respectively (5, 10). The publicly available genome sequencing information makes it easier for legume research community to execute high-throughput research in the areas of transcriptome analysis (14), comparative mapping (15, 16), gene expression studies (17, 18), functional genomics (19), Proteomics (20), etc. Further, being an important legume crop with high economic importance, the role of TFs in various stress conditions is still elusive. Through this work, we believe that our PvTFDB will assist researchers as well as breeders who are working towards crop improvement of legumes family and genome-wide TFs studies.

## Conclusion and future direction

In the present genomic era, it is very challenging to analyse and present the ever increasing data in a meaningful way. PvTFDB is a user-friendly public database, which delivers a range of information about the common bean TFs for public access. This database will reduce the effort of researchers for extracting TFs functional genomic information of common bean. The accessibility of incorporated comprehensive data, including *cis*-regulatory elements, expression profiles and complete TF(s) information is expected to prioritize the further functional analysis of various TFs of interest for breeding. We believe that the information available in the PvTFDB will help basic and applied research studies to better understand the complex regulatory machinery of various stress responses; and further helps engineering of stress tolerant crops. The database will be regularly updated whenever a stress-specific gene expression data is publicly available. Although, there is a TF database available for common bean, PlantTFDB, but our database contains very comprehensive information including tissue-specific gene expression data which is not available in any existed plant TF databases. Hence, we hope that the PvTFDB is novel and useful for research community working on common bean. In future, additional information related to the common bean TFs, the genomic variations, gene co-expression and expression patterns information in different cultivars can also be integrated with PvTFDB to assist the researchers who devoted to improve the common bean crop.

## Supplementary data

Supplementary data are available at *Database* Online. 

Supplementary Data
