# Supplementary material for: PvTFDB: a Phaseolus vulgaris transcription factors database for expediting functional genomics in legumes
Source: Database (Oxford). 2016 Jul 27;2016:baw114. doi: 10.1093/database/baw114 (PMC4962766; doi:10.1093/database/baw114)
Supplement: Supplementary Data [file supp_2016_baw114_index.html]

Supplementary Data 

# PvTFDB: a *Phaseolus vulgaris* transcription factors database for expediting functional genomics in legumes

## Supplementary Data

files

- Supplementary Data - pdf file
