# Supplementary material for: PvTFDB: a Phaseolus vulgaris transcription factors database for expediting functional genomics in legumes
Source: Database (Oxford). 2016 Jul 27;2016:baw114. doi: 10.1093/database/baw114 (PMC4962766; doi:10.1093/database/baw114)
Supplement: Supplementary Data [file supp_baw114_Supplimentary_File1.pdf]

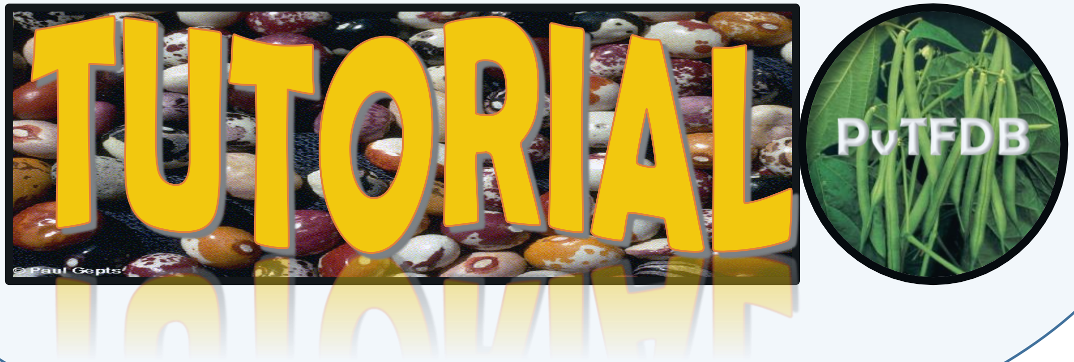
TUTORIAL
© Paul Gepts
PvTFDB

NAVIGATION BAR

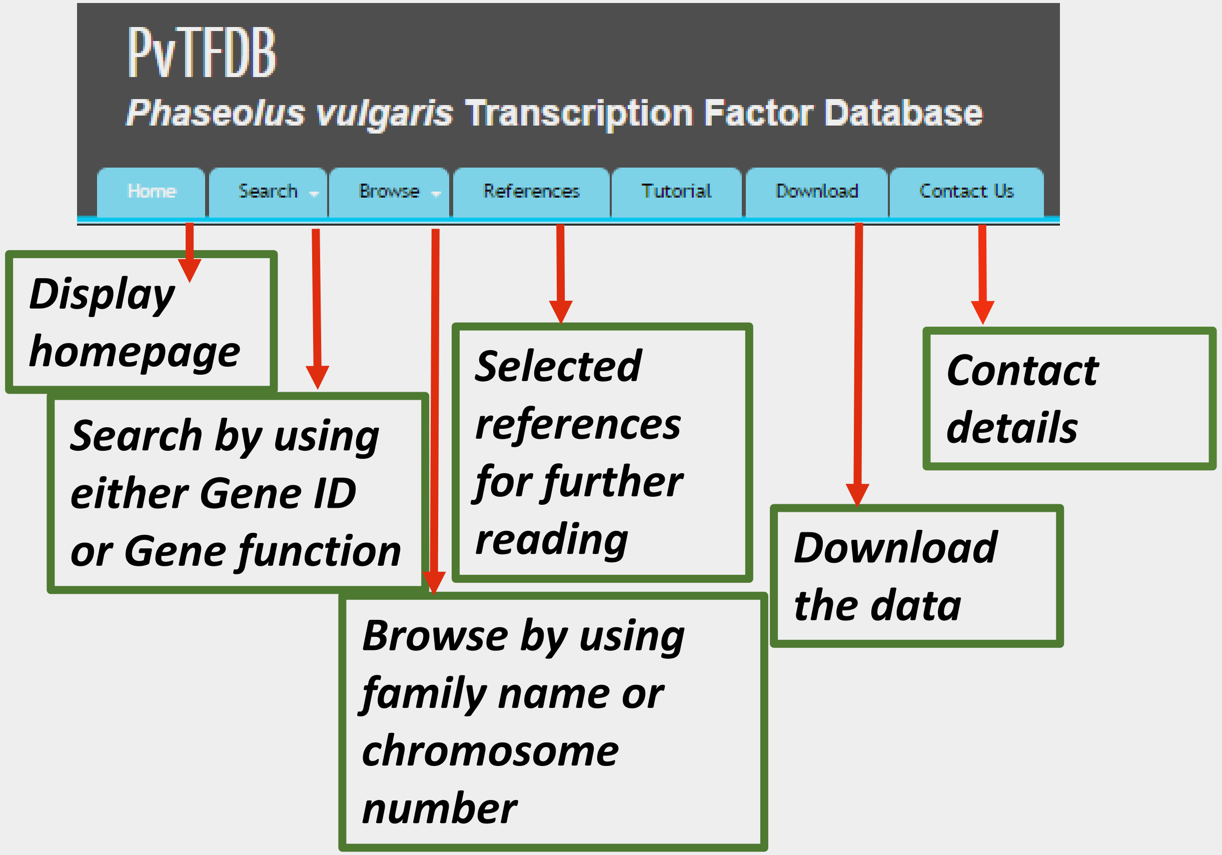

HOMEPAGE

PvTFDB

Phaseolus vulgaris Transcription Factor Database

Home | Search | Browse | References | Tutorial | Download | Contact Us

Welcome to PvTFDB

Phaseolus vulgaris is a herbaceous annual plant grown globally for its eatable dry seed called as beans or green beans for unripe fruit. Its leaf has vegetable properties whereas the straw is used as fodder. It is a member of the legume family *Fabaceae*, well known for its nitrogen-fixing bacteria properties. *Phaseolus vulgaris* is a highly variable climber species with a long history of cultivation. *Phaseolus vulgaris* is one of the most significant food legume in the biosphere. Although this crop is an essential crop to both the developed and developing world as a resources of dietary protein source. It delivers 15% of the protein and 30% of the caloric necessity to the world's population, and symbolises 50% of the grain legumes used worldwide.

The recently published Common bean's genome sequence data (Jeremy, S, et al. 2014) given us an opportunity to develop a comprehensive transcription factors (TF) database, called *Phaseolus vulgaris* Transcription Factors Database (PvTFDB) using various computational methods. PvTFDB is the first online comprehensive TF database developed for the legume crop *Phaseolus vulgaris* L. (Common Bean). This database contains a set of 2370 predicted TFs (Gene models) and classifies them into 49 TF families. In addition, this databse provides comprehensive information at both family and gene level. At family level, this database provides description about the family, list of predicted TFs, phylogenetic tree for entire TF family and tissue specific RNA-Seq based gene expression in the form of heatmap for entire TF family. At gene level, this database provides physical location, sequences (Protein, CDS, Transcript, Genomic and 2kb Upstream sequences), functional annotation (by InterProScan), protein physical properties, mined SSRs along with their primers, mined Cis-elements from 2kb upstream sequences, orthologs in other legume crops along with their protein sequences and tissue specific RNA-Seq expression. This database also provides the researchers to download the entire data used to build the database freely.

The design of PvTFDB database schema follows the "Three-Level-Schema" architecture, is shown in **Figure 1** and developed using open-source softwares, MySQL, Apache and PHP. The PvTFDB schema diagram is shown in **Figure 2**.

To use this database, please follow through the tutorial provided in "Tutorial" tab.

If you use this database, please cite us:
Bhawna, VS Bonthala and Prasad G (2016). PvTFDB: A comprehensive *Phaseolus vulgaris*'s Transcription Factor Database

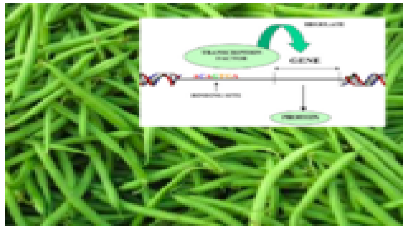

Useful Links

- Common bean's Genome Sequence Data
- Common bean's Genome Sequencing Article
- Common bean's Expression Atlas Database
- Common bean's Expression Atlas Database Article

Visitors Map

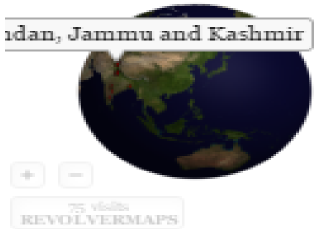

SEARCH BY GENE ID

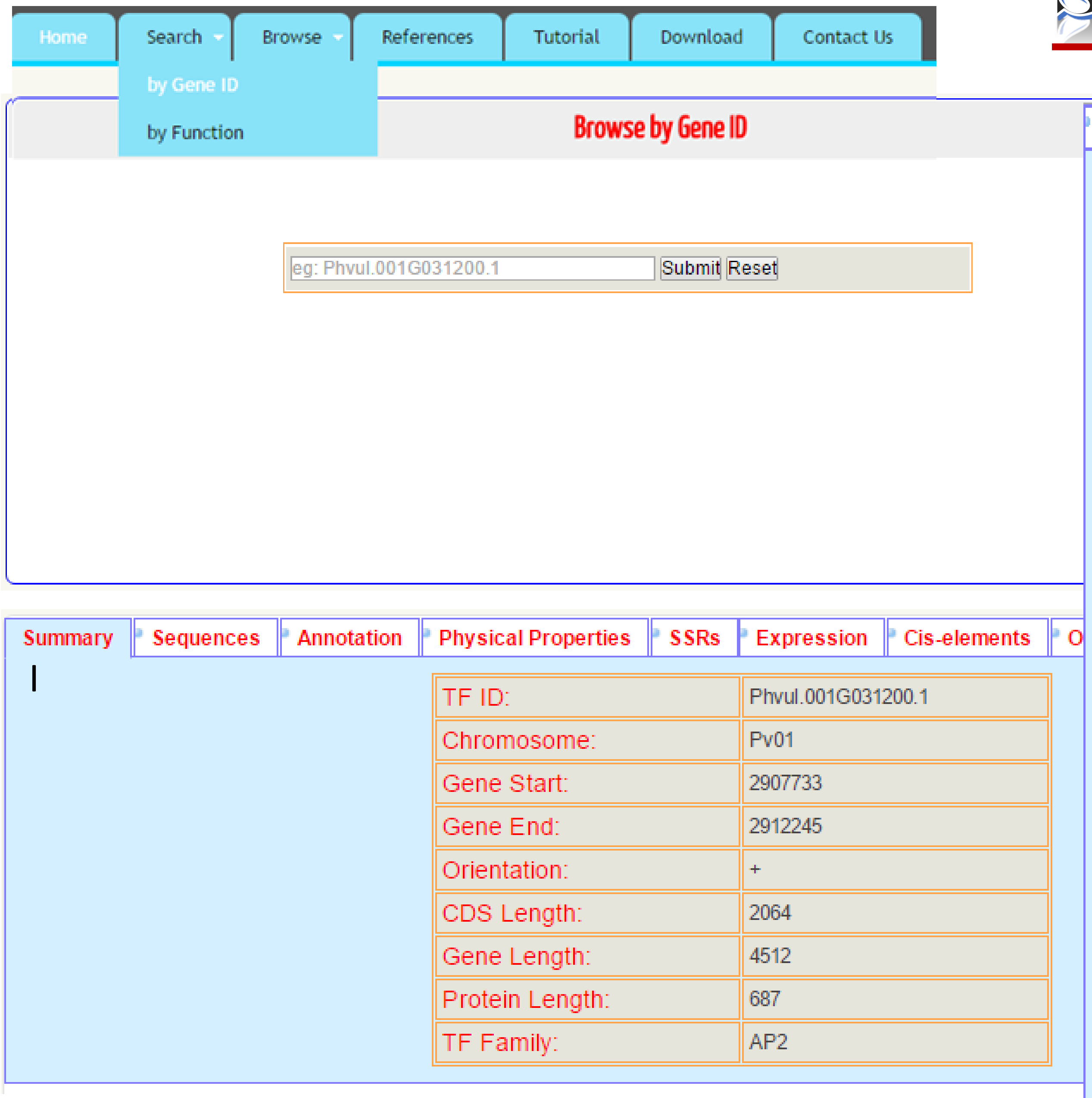

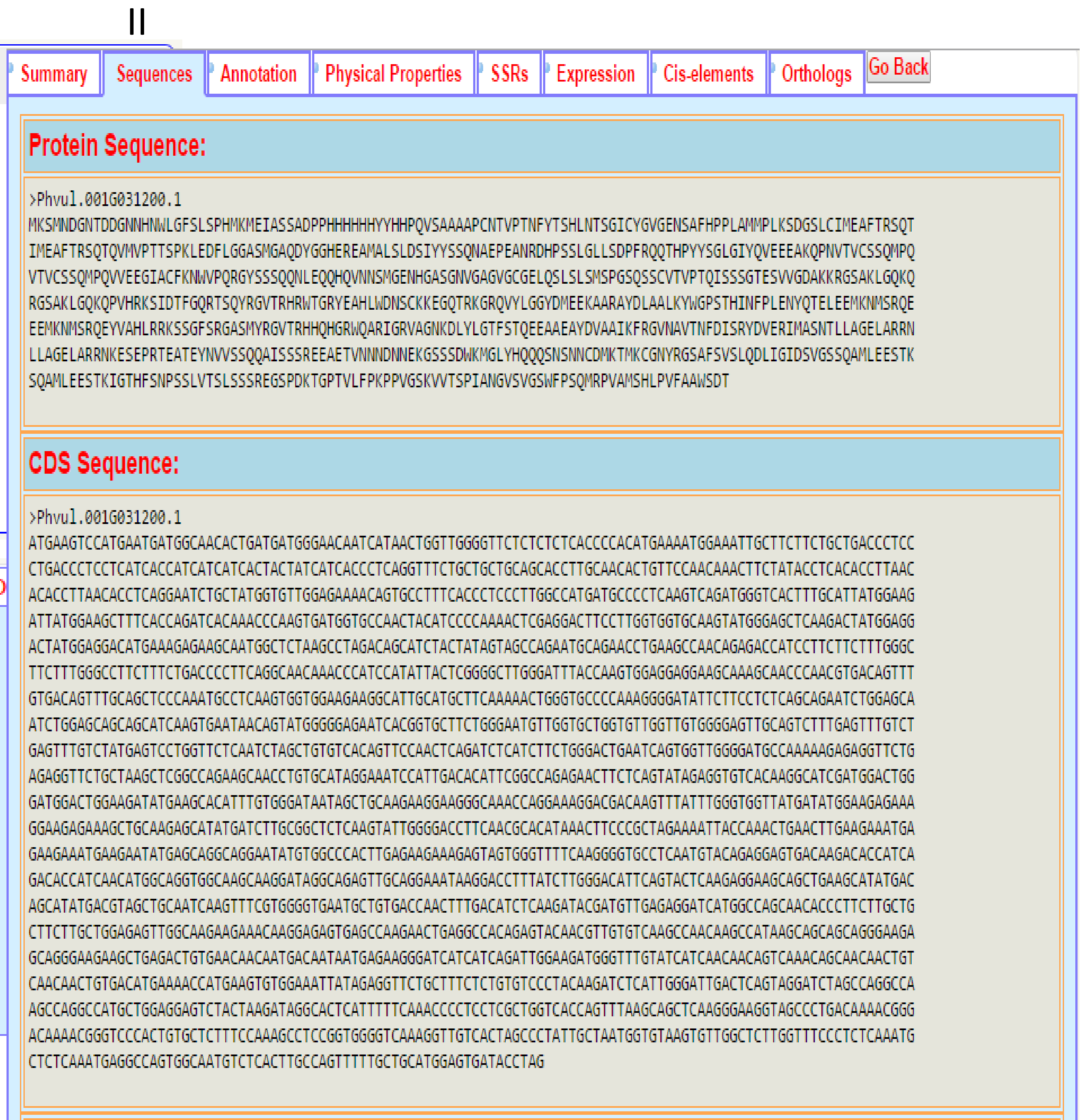

SEARCH BY GENE ID (Cont.)

III

Summary | Sequences | Annotation | Physical Properties | SSRs | Expression | Cis-elements | Orthologs | Go Back

Database	Accession ID	Description	Start	End	E-value
Pfam	PF00847	AP2_domain	429	478	6.9E-12
Pfam	PF00847	AP2_domain	325	384	1.1E-13
SMART	SM00380	DNA-binding_domain_in_plant_proteins_such_as_APETALA2_and_EREBPs	428	492	4.9E-34
SMART	SM00380	DNA-binding_domain_in_plant_proteins_such_as_APETALA2_and_EREBPs	326	398	1.8E-28
PRINTS	PR00367	Ethylene_responsive_element_binding_protein_signature	327	338	3.4E-6
PRINTS	PR00367	Ethylene_responsive_element_binding_protein_signature	468	488	3.4E-6
InterProScan	IPR001471	AP2/ERF_domain			
InterProScan	IPR016177	DNA-binding_domain			
ProSiteProfiles	PS51032	[illegible]	[illegible]	[illegible]	[illegible]
ProSiteProfiles	PS51032	[illegible]	[illegible]	[illegible]	[illegible]
SUPERFAMILY	SSF54171	[illegible]	[illegible]	[illegible]	[illegible]
SUPERFAMILY	SSF54171	[illegible]	[illegible]	[illegible]	[illegible]
Gene3D	G3DSA:3.30.730.10	[illegible]	[illegible]	[illegible]	[illegible]
Gene3D	G3DSA:3.30.730.10	[illegible]	[illegible]	[illegible]	[illegible]
PANTHER	PTHR32467:SF0	[illegible]	[illegible]	[illegible]	[illegible]
PANTHER	PTHR32467	[illegible]	[illegible]	[illegible]	[illegible]
GO	GO:0006355	[illegible]	[illegible]	[illegible]	[illegible]
GO	GO:0003700	[illegible]	[illegible]	[illegible]	[illegible]
GO	GO:0007275	[illegible]	[illegible]	[illegible]	[illegible]

IV

Summary | Sequences | Annotation | Physical Properties | SSRs | Expression | Cis-elements | Orthologs | Go Back

Molecular Weight (Da):	75431.6671
Aromaticity:	0.077
Instability Index:	49.51 [Stable < 40 > Unstable]
Isoelectric Point (pI):	6.639
Fraction of Amino Acids:	
in Helix:	0.2212
in Turn:	0.3158
in Sheet:	0.2241

SEARCH BY GENE ID (Cont.)

V

Summary | Sequences | Annotation | Physical Properties | SSRs | Expression | Cis-elements | Orthologs | Go Back

SSR Motif	Motif Length	Motif Repeat	Motif Repeat Length	SSR Motif Sequence	Get
AGC	3	3	9	AGCAGCAGC	Primers
ATC	3	3	9	ATCATCATC	Primers
CAT	3	3	9	CATCATCAT	Primers
CTG	3	3	9	CTGCTGCTG	Primers
CTT	3	3	9	CTTCTTCTT	Primers
GGA	3	3	9	GGAGGAGGA	Primers

Primer Details:

Forward Primer	Start	Length	Tm^0 C	GC %	Product Size	Reverse Primer	Start	Length	Tm^0 C	GC %
ATTCTTCCTCTCAGCAGAATC	683	21	55.25	42.86	146	TTGAGAACCAGGACTCATAGA	828	21	54.92	42.86
ATTCTTCCTCTCAGCAGAATC	683	21	55.25	42.86	152	GCTAGATTGAGAACCAGGACT	834	21	55.21	47.62
ATTCTTCCTCTCAGCAGAATC	683	21	55.25	42.86	149	AGATTGAGAACCAGGACTCAT	831	21	55.29	42.86
GCAAGAAGAAACAAGGAGAGT	1519	21	55.41	42.86	145	AATCTGATGATGATCCCTTCT	1663	21	55.13	38.1
GATACGATGTTGAGAGGATCA	1466	21	55.13	42.86	163	TTGTTCACAGTCTCAGCTTCT	1628	21	55.24	42.86
CAAGAAGAAACAAGGAGAGTG	1520	21	54.31	42.86	144	AATCTGATGATGATCCCTTCT	1663	21	55.13	38.1

SEARCH BY GENE ID (Cont.)

VI

Summary | Sequences | Annotation | Physical Properties | SSRs | Expression | Cis-elements | Orthologs | Go Back

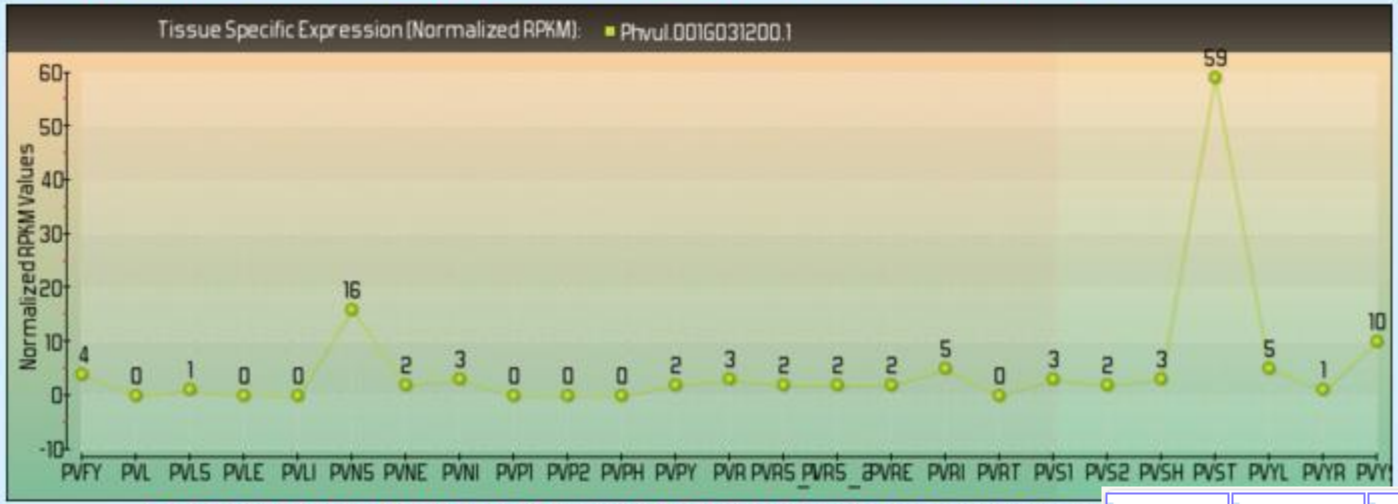

For complete details about this heatmap of expression data, please go through The Common Bean Gene

VII

Summary | Sequences | Annotation | Physical Properties | SSRs | Expression | Cis-elements | Orthologs | Go Back

Site Name	Location	Strand	Signal Sequence	Site ID
ARR1AT	9	-	NGATT	S000454
ARR1AT	14	-	NGATT	S000454
POLASIG2	27	+	AATTAAA	S000081
SEF4MOTIFGM7S	30	-	RTTTTTR	S000103
CACTFTPPCA1	37	+	YACT	S000449
WBOXNTCHN48	39	+	CTGACY	S000508
WBOXHVISO1	40	+	TGACT	S000442
WRKY71OS	40	+	TGAC	S000447
WBOXNTERF3	40	+	TGACY	S000457
TATABOX5	45	-	TTATTT	S000203
POLASIG3	46	+	AATAAT	S000088

VIII

Summary | Sequences | Annotation | Physical Properties | SSRs | Expression | Cis-elements | Orthologs | Go Back

Plant	Ortholog ID	Identity (%)	Start	End	E-Value
Lotus japonicus	Lj0g3v0070889.2	95.00	213	232	1e-05
Lotus japonicus	Lj0g3v0070889.1	95.00	158	177	5e-06
AdzukiBean	Vang01g18250.1	91.19	1	690	0.0
Lotus japonicus	Lj3g3v2612440.1	86.92	1	107	1e-59
Pigeonpea	C.cajan_04959	86.46	147	503	0.0
Lotus japonicus	Lj2g3v1455120.1	84.78	211	256	3e-21
Soybean	Glyma09g33241.1	84.78	211	256	2e-21
Pigeonpea	C.cajan_18251	84.69	200	395	2e-114
Soybean	Glyma14g10130.2	84.36	1	599	0.0
Soybean	Glyma0041s50.1	84.34	1	529	0.0
Mungbean	Vradi06g14410.1	82.31	1	626	0.0
Lotus japonicus	Lj4g3v2120330.1	81.08	1	111	5e-56
Soybean	Glyma17g07010.1	80.98	134	337	4e-117
Soybean	Glyma13g00950.1	80.98	130	333	2e-117
Pigeonpea	C.cajan_03656	80.79	132	333	1e-116
Lotus japonicus	Lj1g3v2841310.1	80.36	133	188	1e-24

SEARCH BY FUNCTION

Home | Search | Browse | References | Tutorial | Download | Contact Us

by Gene ID

by Function

Search by Function

GO ID:	GO:0003677	Submit Reset
Pfam ID:	eg: PF00847	Submit Reset
SMART ID:	eg: SM01019	
InterProScan ID:	eg: IPR003340	
ProSiteProfiles ID:	eg: PS50863	

Summary | Sequences | Annotation | Physical Properties | SSRs | Expression | Cis-elements

TF ID:	Phvul.001G008200.1
Chromosome:	Pv01
Gene Start:	704238
Gene End:	709592
Orientation:	+
CDS Length:	2814
Gene Length:	5354
Protein Length:	937
TF Family:	ARF

There are 945 Transcription Factors are present with GO ID: GO:0003677

Phvul.001G008200.1 Phvul.001G011000.1 Phvul.001G011000.2 Phvul.001G011100.1 Phvul.001G018400.1
Phvul.001G018500.1 Phvul.001G018500.2 Phvul.001G018500.3 Phvul.001G018500.4 Phvul.001G023400.1
Phvul.001G023600.1 Phvul.001G032200.1 Phvul.001G032200.2 Phvul.001G034200.1 Phvul.001G059900.1
Phvul.001G059900.2 Phvul.001G062100.1 Phvul.001G068200.1 Phvul.001G072200.1 Phvul.001G080800.1
Phvul.001G086800.1 Phvul.001G091100.1 Phvul.001G091400.1 Phvul.001G100200.1 Phvul.001G100500.1
Phvul.001G106400.1 Phvul.001G106700.1 Phvul.001G117200.1 Phvul.001G126400.1 Phvul.001G141000.1
Phvul.001G143500.1 Phvul.001G161700.1 Phvul.001G175700.1 Phvul.001G176100.1 Phvul.001G184800.1
Phvul.001G184900.1 Phvul.001G186400.1 Phvul.001G192000.1 Phvul.001G193100.1 Phvul.001G195800.1
Phvul.001G196100.1 Phvul.001G196900.1 Phvul.001G202000.1 Phvul.001G224900.1 Phvul.001G228600.1
Phvul.001G241000.1 Phvul.001G254000.1 Phvul.001G262600.1 Phvul.001G263000.1 Phvul.001G263400.1
Phvul.001G263400.2 Phvul.002G008600.1 Phvul.002G010300.1 Phvul.002G022000.1 Phvul.002G024800.1
Phvul.002G027200.1 Phvul.002G048200.1 Phvul.002G048200.2 Phvul.002G049800.1 Phvul.002G056300.1
Phvul.002G061000.1 Phvul.002G061000.2 Phvul.002G064100.1 Phvul.002G064100.2 Phvul.002G067200.1
Phvul.002G084000.1 Phvul.002G085700.1 Phvul.002G088600.1 Phvul.002G094100.1 Phvul.002G095800.1
Phvul.002G100800.1 Phvul.002G100800.2 Phvul.002G109400.1 Phvul.002G110900.1 Phvul.002G111600.1
Phvul.002G112200.1 Phvul.002G112200.2 Phvul.002G114500.1 Phvul.002G114500.2 Phvul.002G137000.1
Phvul.002G143900.1 Phvul.002G143900.2 Phvul.002G144900.1 Phvul.002G147600.1 Phvul.002G153300.1
Phvul.002G159000.1 Phvul.002G170200.1 Phvul.002G170400.1 Phvul.002G170600.1 Phvul.002G171600.1
Phvul.002G172300.1 Phvul.002G172300.2 Phvul.002G172800.1 Phvul.002G177600.1 Phvul.002G183400.1
Phvul.002G187900.1 Phvul.002G194600.1 Phvul.002G197500.1 Phvul.002G206300.1 Phvul.002G209300.1
Phvul.002G212400.1 Phvul.002G215500.1 Phvul.002G224900.1 Phvul.002G226100.1 Phvul.002G229100.1
Phvul.002G229800.1 Phvul.002G229800.2 Phvul.002G230100.1 Phvul.002G230200.1 Phvul.002G230300.1

SEARCH BY FUNCTION (Cont.)

Search by Function

GO ID:	eg: GO:0003677	Submit Reset
Pfam ID:	PF00847	Submit Reset
SMART ID:	eg: SM01019	Submit Reset
InterProScan ID:	eg: IPR003340	Submit Reset
ProSiteProfiles ID:	eg: PS50863	Submit Reset

Summary | Sequences | Annotation | Physical Properties | SSRs | Expression | Cis-elements | Orthologs | Go Back

TF ID:	Phvul.002G009100.1
Chromosome:	Pv02
Gene Start:	1058028
Gene End:	1061555
Orientation:	-
CDS Length:	1797
Gene Length:	3527
Protein Length:	598
TF Family:	AP2

There are 220 Transcription Factors are present with Pfam ID: PF00847

Phvul.002G009100.1 Phvul.002G009100.1 Phvul.002G056800.1 Phvul.002G267800.1 Phvul.005G138300.1
Phvul.005G138300.1 Phvul.008G019600.1 Phvul.003G069000.1 Phvul.003G069000.1 Phvul.009G251600.1
Phvul.005G126600.1 Phvul.005G170600.1 Phvul.011G071100.1 Phvul.011G071100.1 Phvul.007G102800.1
Phvul.007G102800.1 Phvul.007G082000.1 Phvul.007G082000.2 Phvul.010G035200.1 Phvul.002G035900.1
Phvul.001G160300.1 Phvul.002G009100.2 Phvul.002G009100.2 Phvul.007G272800.1 Phvul.005G126300.1
Phvul.006G047800.1 Phvul.009G123300.1 Phvul.008G246000.1 Phvul.008G043500.1 Phvul.008G043500.1
Phvul.001G023700.1 Phvul.003G165000.1 Phvul.009G074300.1 Phvul.009G074300.1 Phvul.007G241600.1
Phvul.002G055800.1 Phvul.005G105200.1 Phvul.008G046400.1 Phvul.010G092300.1 Phvul.004G081200.1
Phvul.001G137500.1 Phvul.010G050600.1 Phvul.008G131500.2 Phvul.006G163100.1 Phvul.002G163700.1
Phvul.010G130200.1 Phvul.010G130200.1 Phvul.002G295700.1 Phvul.003G212700.1 Phvul.004G169800.1
Phvul.007G128100.1 Phvul.001G031200.1 Phvul.001G031200.1 Phvul.002G016900.1 Phvul.002G016900.1
Phvul.003G111800.1 Phvul.003G111800.1 Phvul.010G050700.1 Phvul.002G016700.1 Phvul.001G131300.1
Phvul.007G135300.1 Phvul.007G128000.1 Phvul.003G232600.1 Phvul.003G232600.1 Phvul.010G114900.1
Phvul.001G160400.1 Phvul.003G180000.1 Phvul.008G159400.1 Phvul.002G153900.1 Phvul.002G149500.1
Phvul.011G058000.1 Phvul.011G058000.1 Phvul.004G122000.1 Phvul.008G271100.1 Phvul.009G137000.1
Phvul.001G157600.1 Phvul.011G107800.1 Phvul.011G107800.2 Phvul.008G172200.1 Phvul.003G223600.1
Phvul.001G187100.1 Phvul.003G035500.1 Phvul.010G124700.1 Phvul.009G013200.1 Phvul.007G255100.1

SEARCH BY FUNCTION (Cont.)

Search by Function

GO ID:	eg: GO:0003677	Submit Reset
Pfam ID:	eg: PF00847	Submit Reset
SMART ID:	SM01019	Submit Reset
InterProScan ID:	eg: IPR003340	Submit Reset
ProSiteProfiles ID:	eg: PS50863	Submit Reset

There are 123 Transcription Factors are present with SMART ID: SM01019

Phvul.006G181200.1 Phvul.001G018500.2 Phvul.001G018500.2 Phvul.001G018500.2 Phvul.001G018500.3
Phvul.001G018500.3 Phvul.001G018500.3 Phvul.002G229800.2 Phvul.007G102800.1 Phvul.007G102800.1
Phvul.003G128800.1 Phvul.009G064100.1 Phvul.006G100700.1 Phvul.006G100700.1 Phvul.006G079300.2
Phvul.006G079300.1 Phvul.010G120900.3 Phvul.010G120900.1 Phvul.010G120900.2 Phvul.010G011600.1
Phvul.011G050800.1 Phvul.011G050800.1 Phvul.001G018500.4 Phvul.001G018500.4 Phvul.001G018500.4
Phvul.001G018500.1 Phvul.001G018500.1 Phvul.001G018500.1 Phvul.008G244300.1 Phvul.002G282200.1
Phvul.011G073600.2 Phvul.001G008200.1 Phvul.007G022900.1 Phvul.007G022900.1 Phvul.009G169300.1
Phvul.006G114300.1 Phvul.007G149000.1 Phvul.007G019500.1 Phvul.007G019500.1 Phvul.003G011300.1
Phvul.003G011300.1 Phvul.011G050700.2 Phvul.011G050700.2 Phvul.011G050700.1 Phvul.011G050700.1
Phvul.009G260100.1 Phvul.003G111800.1 Phvul.003G111800.1 Phvul.003G033400.1 Phvul.003G033400.1
Phvul.003G033400.1 Phvul.005G134500.2 Phvul.001G193100.1 Phvul.001G018400.1 Phvul.001G018400.1
Phvul.006G084200.2 Phvul.002G229800.1 Phvul.002G177600.1 Phvul.008G197600.1 Phvul.008G220000.1
Phvul.011G080100.1 Phvul.005G136900.1 Phvul.008G247500.1 Phvul.002G233600.1 Phvul.008G242400.1
Phvul.003G075800.1 Phvul.007G002900.1 Phvul.007G002900.1 Phvul.003G007600.1 Phvul.003G007600.1
Phvul.003G007600.2 Phvul.003G007600.2 Phvul.011G050600.2 Phvul.011G050600.2 Phvul.008G118400.1
Phvul.011G050600.1 Phvul.011G050600.1 Phvul.008G140200.1 Phvul.011G073600.1 Phvul.008G118400.2
Phvul.003G007500.1 Phvul.003G007500.1 Phvul.007G041900.1 Phvul.007G041900.1 Phvul.007G041900.1
Phvul.009G169300.2 Phvul.007G019600.1 Phvul.007G019600.1 Phvul.011G050500.1 Phvul.001G202000.1
Phvul.005G000700.1 Phvul.005G000700.1 Phvul.010G070200.2 Phvul.010G070200.1 Phvul.007G019400.1
Phvul.007G019400.1 Phvul.007G019400.1 Phvul.008G122100.1 Phvul.009G169300.5 Phvul.009G026200.1
Phvul.005G134500.1 Phvul.003G007600.3 Phvul.003G007600.3 Phvul.003G007600.4 Phvul.003G007600.4
Phvul.007G230700.1 Phvul.009G161900.1 Phvul.006G191700.1 Phvul.004G177600.1 Phvul.008G168700.1
Phvul.006G084200.1 Phvul.003G290200.1 Phvul.008G140100.1 Phvul.007G019700.1 Phvul.007G019700.1
Phvul.007G095000.1 Phvul.001G086800.1 Phvul.003G007400.1 Phvul.003G007400.1 Phvul.009G224800.1
Phvul.003G244400.1 Phvul.006G221500.1 Phvul.003G011300.2

Summary | Sequences | Annotation | Physical Properties | SSRs | Expression | Cis-elements | Orthologs | Go

TF ID:	Phvul.006G181200.1
Chromosome:	Pv06
Gene Start:	29058029
Gene End:	29064102
Orientation:	+
CDS Length:	2205
Gene Length:	6073
Protein Length:	734
TF Family:	ARF

SEARCH BY FUNCTION (Cont.)

Search by Function

GO ID:	eg: GO:0003677	Submit Reset
Pfam ID:	eg: PF00847	Submit Reset
SMART ID:	eg: SM01019	Submit Reset
InterProScan ID:	IPR003340	Submit Reset
ProSiteProfiles ID:	eg: PS50863	Submit Reset

Annotation | Physical Properties | SSRs | Expression | Cis-elements | Orthologs | Go Back

TF ID:	Phvul.006G181200.1
Chromosome:	Pv06
Gene Start:	29058029
Gene End:	29064102
Orientation:	+
CDS Length:	2205
Gene Length:	6073
Protein Length:	734
TF Family:	ARF

There are 99 Transcription Factors are present with InterProScan ID: IPR003340

Phvul.006G181200.1 Phvul.001G018500.2 Phvul.001G018500.3 Phvul.004G169400.1 Phvul.002G229800.2
Phvul.007G102800.1 Phvul.007G102800.1 Phvul.003G128800.1 Phvul.009G064100.1 Phvul.006G100700.1
Phvul.006G079300.2 Phvul.006G079300.1 Phvul.010G120900.3 Phvul.010G120900.1 Phvul.010G120900.2
Phvul.010G011600.1 Phvul.011G050800.1 Phvul.001G018500.4 Phvul.001G018500.1 Phvul.008G244300.1
Phvul.002G282200.1 Phvul.011G073600.2 Phvul.001G008200.1 Phvul.007G022900.1 Phvul.005G031300.1
Phvul.009G169300.1 Phvul.006G114300.1 Phvul.007G149000.1 Phvul.007G019500.1 Phvul.003G011300.1
Phvul.011G050700.2 Phvul.011G050700.1 Phvul.009G260100.1 Phvul.003G111800.1 Phvul.003G111800.1
Phvul.003G033400.1 Phvul.005G134500.2 Phvul.001G193100.1 Phvul.001G018400.1 Phvul.006G084200.2
Phvul.002G229800.1 Phvul.002G177600.1 Phvul.008G197600.1 Phvul.008G220000.1 Phvul.011G080100.1
Phvul.005G136900.1 Phvul.009G169300.6 Phvul.008G247500.1 Phvul.002G233600.1 Phvul.008G242400.1
Phvul.003G075800.1 Phvul.007G002900.1 Phvul.007G002900.1 Phvul.003G007600.1 Phvul.003G007600.2
Phvul.011G050600.2 Phvul.008G118400.1 Phvul.011G050600.1 Phvul.008G140200.1 Phvul.004G169500.1
Phvul.011G073600.1 Phvul.008G118400.2 Phvul.003G007500.1 Phvul.007G041900.1 Phvul.009G169300.2
Phvul.007G019600.1 Phvul.011G050500.1 Phvul.001G202000.1 Phvul.005G000700.1 Phvul.010G070200.2
Phvul.010G070200.1 Phvul.007G019400.1 Phvul.008G122100.1 Phvul.009G169300.5 Phvul.009G026200.1
Phvul.005G134500.1 Phvul.003G007600.3 Phvul.003G007600.4 Phvul.007G230700.1 Phvul.009G161900.1
Phvul.009G169300.4 Phvul.006G191700.1 Phvul.004G177600.1 Phvul.008G168700.1 Phvul.006G084200.1
Phvul.003G290200.1 Phvul.009G169300.3 Phvul.008G140100.1 Phvul.002G100800.2 Phvul.007G019700.1
Phvul.007G095000.1 Phvul.001G086800.1 Phvul.003G007400.1 Phvul.009G224800.1 Phvul.003G244400.1
Phvul.002G100800.1 Phvul.006G221500.1 Phvul.003G011300.2

SEARCH BY FUNCTION (Cont.)

Search by Function

GO ID:	eg: GO:0003677	Submit Reset
Pfam ID:	eg: PF00847	Submit Reset
SMART ID:	eg: SM01019	Submit Reset
InterProScan ID:	eg: IPR003340	Submit R
ProSiteProfiles ID:	PS50863	Submit R

Summary | Sequences | Annotation | Physical Properties | SSRs | Expression | Cis-elements | Ortholo

TF ID:	Phvul.006G181200.1
Chromosome:	Pv06
Gene Start:	29058029
Gene End:	29064102
Orientation:	+
CDS Length:	2205
Gene Length:	6073
Protein Length:	734
TF Family:	ARF

There are 136 Transcription Factors are present with PrositeProfile ID: PS50863

Phvul.006G181200.1 Phvul.001G018500.2 Phvul.001G018500.2 Phvul.001G018500.2 Phvul.001G018500.3
Phvul.001G018500.3 Phvul.001G018500.3 Phvul.004G169400.1 Phvul.002G229800.2 Phvul.007G102800.1
Phvul.007G102800.1 Phvul.003G128800.1 Phvul.009G064100.1 Phvul.006G100700.1 Phvul.006G100700.1
Phvul.006G079300.2 Phvul.006G079300.1 Phvul.010G120900.3 Phvul.010G120900.1 Phvul.010G120900.2
Phvul.010G011600.1 Phvul.011G050800.1 Phvul.011G050800.1 Phvul.001G018500.4 Phvul.001G018500.4
Phvul.001G018500.4 Phvul.001G018500.1 Phvul.001G018500.1 Phvul.001G018500.1 Phvul.008G244300.1
Phvul.002G282200.1 Phvul.011G073600.2 Phvul.001G008200.1 Phvul.007G022900.1 Phvul.007G022900.1
Phvul.005G031300.1 Phvul.009G169300.1 Phvul.009G169300.1 Phvul.006G114300.1 Phvul.007G149000.1
Phvul.007G019500.1 Phvul.007G019500.1 Phvul.003G011300.1 Phvul.003G011300.1 Phvul.011G050700.2
Phvul.011G050700.2 Phvul.011G050700.1 Phvul.011G050700.1 Phvul.009G260100.1 Phvul.003G111800.1
Phvul.003G111800.1 Phvul.003G033400.1 Phvul.003G033400.1 Phvul.003G033400.1 Phvul.005G134500.2
Phvul.001G193100.1 Phvul.001G018400.1 Phvul.001G018400.1 Phvul.006G084200.2 Phvul.002G229800.1
Phvul.002G177600.1 Phvul.008G197600.1 Phvul.008G220000.1 Phvul.008G220000.1 Phvul.011G080100.1
Phvul.005G136900.1 Phvul.009G169300.6 Phvul.008G247500.1 Phvul.002G233600.1 Phvul.008G242400.1
Phvul.003G075800.1 Phvul.007G002900.1 Phvul.007G002900.1 Phvul.003G007600.1 Phvul.003G007600.1
Phvul.003G007600.2 Phvul.003G007600.2 Phvul.011G050600.2 Phvul.011G050600.2 Phvul.008G118400.1
Phvul.011G050600.1 Phvul.011G050600.1 Phvul.004G169500.1 Phvul.011G073600.1 Phvul.008G118400.2
Phvul.003G007500.1 Phvul.003G007500.1 Phvul.007G041900.1 Phvul.007G041900.1 Phvul.007G041900.1
Phvul.009G169300.2 Phvul.009G169300.2 Phvul.007G019600.1 Phvul.007G019600.1 Phvul.011G050500.1
Phvul.001G202000.1 Phvul.005G000700.1 Phvul.005G000700.1 Phvul.010G070200.2 Phvul.010G070200.1
Phvul.007G019400.1 Phvul.007G019400.1 Phvul.007G019400.1 Phvul.008G122100.1 Phvul.009G169300.5
Phvul.009G026200.1 Phvul.005G134500.1 Phvul.003G007600.3 Phvul.003G007600.3 Phvul.003G007600.4
Phvul.003G007600.4 Phvul.007G230700.1 Phvul.009G161900.1 Phvul.009G169300.4 Phvul.009G169300.4
Phvul.006G191700.1 Phvul.004G177600.1 Phvul.008G168700.1 Phvul.006G084200.1 Phvul.003G290200.1
Phvul.009G169300.3 Phvul.009G169300.3 Phvul.008G140100.1 Phvul.002G100800.2 Phvul.007G019700.1
Phvul.007G019700.1 Phvul.007G095000.1 Phvul.003G007400.1 Phvul.003G007400.1 Phvul.009G224800.1
Phvul.003G244400.1 Phvul.002G100800.1 Phvul.006G221500.1 Phvul.003G011300.2

BROWSE BY FAMILY

Home | Search | Browse | References | Tutorial | Download | Contact Us

by Family

by Chromosome

Browse by Family

AP2 (25)	ARF (31)	ARR-B (60)	B3 (61)	BBR-BPC (9)
BES1 (7)	C2H2 (211)	C3H (95)	CAMTA (13)	CO-like (18)
CPP (6)	DBB (12)	Dof (45)	E2F/DP (14)	EIL (7)
ERF (163)	FAR1 (32)	GATA (40)	GRAS (63)	GRF (23)
GeBP (5)	HB (142)	HSF (35)	LBD (53)	LFY (1)
LSD (7)	M-type (50)	MIKC (42)	MYB (145)	MYB_related (157)
NAC (106)	NF-X1 (2)	NF-Y (60)	Nin-like (15)	NOZZLE (2)
RAV (3)	S1Fa-like (3)	SAP (57)	SBP (29)	SRS (13)
STAT (3)	TCP (31)	Trihelix (47)	WRKY (102)	Whirly (4)
YABBY (9)	ZF-HD (19)	bHLH (191)	bZIP (102)	

BROWSE BY FAMILY (CONT.)

FAMILY DESCRIPTION

Description | List of TFs | Phylogenetic Tree | Heatmap | Click here to Go Back

Family: AP2

Family Description:

The AP2/ERF superfamily is defined by the AP2/ERF domain, which consists of about 60 to 70 amino acids and is involved in DNA binding. These three families have been defined as follows. The AP2 family proteins contain two repeated AP2/ERF domains, the ERF family proteins contain a single AP2/ERF domain, and the RAV family proteins contain a B3 domain, which is a DNA-binding domain conserved in other plant-specific transcription factors, in addition to the single AP2/ERF domain. It has been demonstrated that the AP2/ERF proteins have important functions in the transcriptional regulation of a variety of biological processes related to growth and development, as well as various responses to environmental stimuli. Genes in the AP2 family have been shown to participate in the regulation of developmental processes, e.g. flower development, spikelet meristem determinacy, leaf epidermal cell identity, and embryo development. Using an in vitro selection procedure, the DNA binding specificity of the two AP2 repeat containing protein ANT was found to be 5'-gCAC(A/G)N(A/T)TcCC(a/g)ANG(c/t)-3'. This consensus site is much longer than sites recognized by proteins containing a single AP2 repeat and neither AP2 repeat of ANT was alone capable of binding to the selected sequences, suggesting that both AP2 repeats make DNA contacts.

Reference: 16407444

BROWSE BY FAMILY (CONT.)

Description | List of TFs | Phylogenetic Tree | Heatmap | Click here to Go Back

25 Transcription Factors are present in AP2

Phvul.001G031200.1 Phvul.001G174400.1 Phvul.002G009100.1 Phvul.002G009100.2 Phvul.002G016900.1 Phvul.002G146400.1 Phvul.003G069000.1 Phvul.003G069000.2 Phvul.003G144500.1 Phvul.003G153100.1 Phvul.003G232600.1 Phvul.003G241900.1 Phvul.003G288500.1 Phvul.005G138300.1 Phvul.005G183700.1 Phvul.008G043500.1 Phvul.008G052000.1 Phvul.008G185400.1 Phvul.008G253600.1 Phvul.009G074300.1 Phvul.009G206500.1 Phvul.010G130200.1 Phvul.011G187400.1 Phvul.011G058000.1 Phvul.011G071100.1

Description | List of TFs | Phylogenetic Tree | Heatmap | Click here to Go Back

The phylogenetic Tree was created using MEGA software

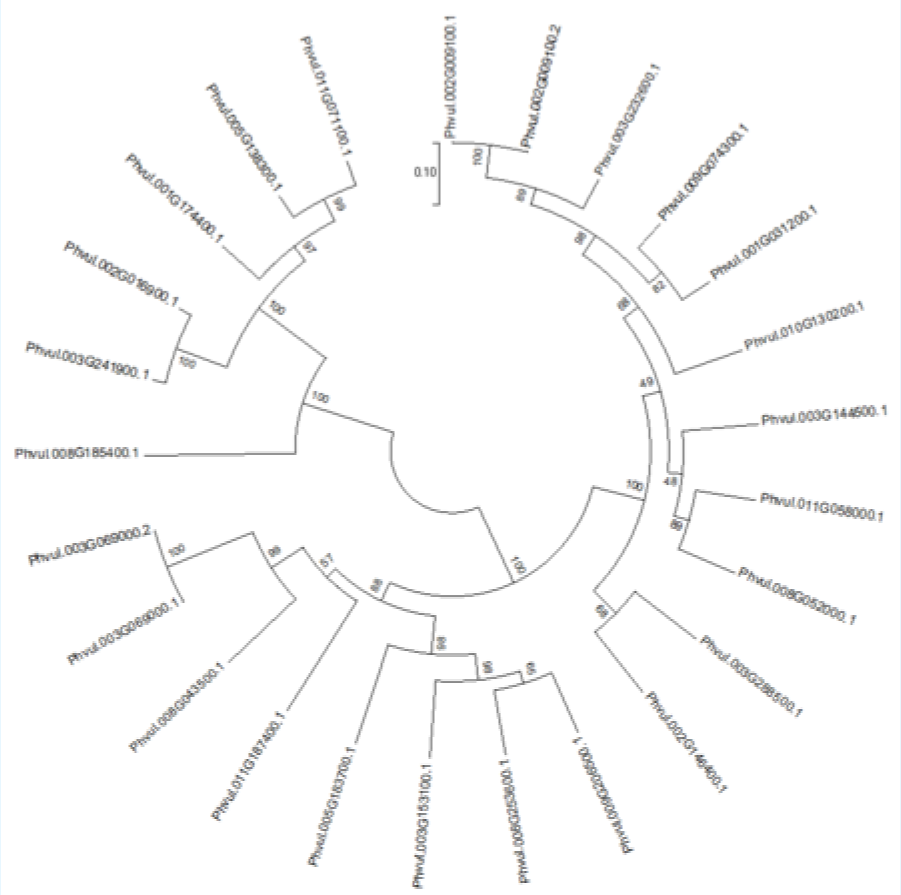

Description | List of TFs | Phylogenetic Tree | Heatmap | Click here to Go Back

Tissue Specific Expression of Entire TF Family using normalized RPKM values.

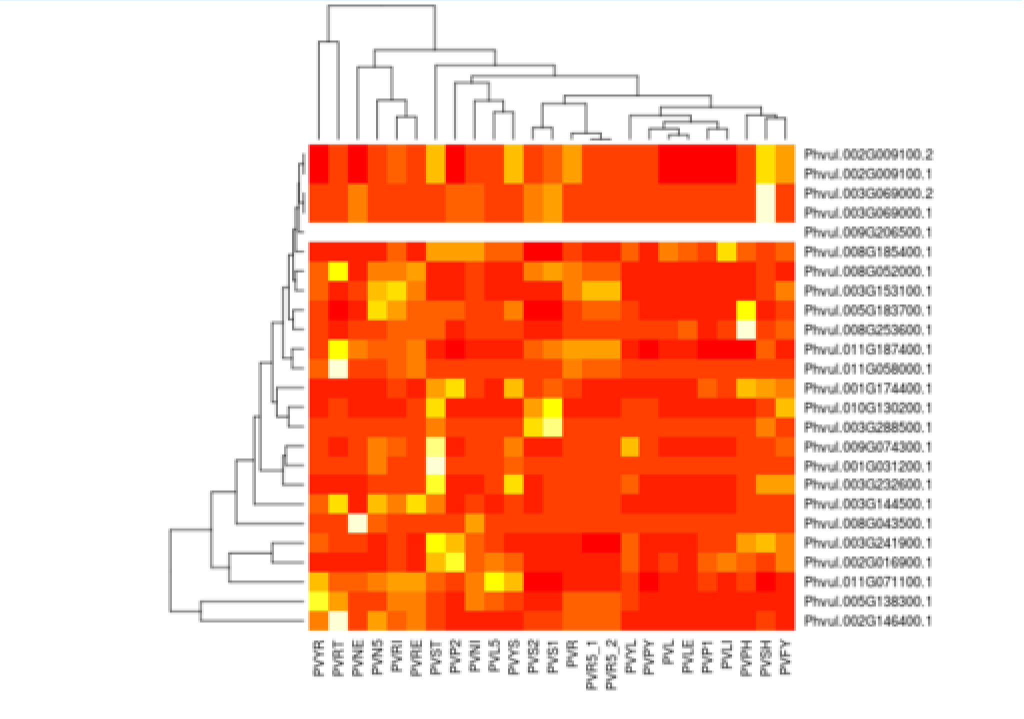

For complete details about this heatmap of expression data, please go through The Common Bean Gene

BROWSE BY CHROMOSOME

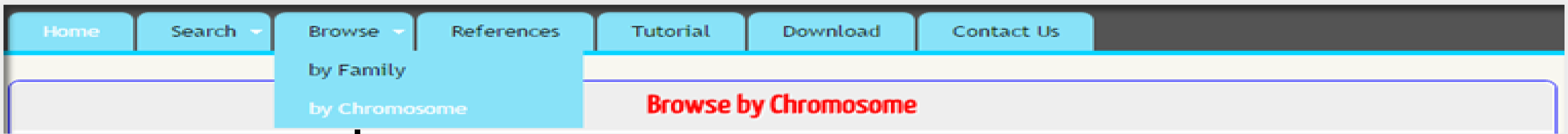

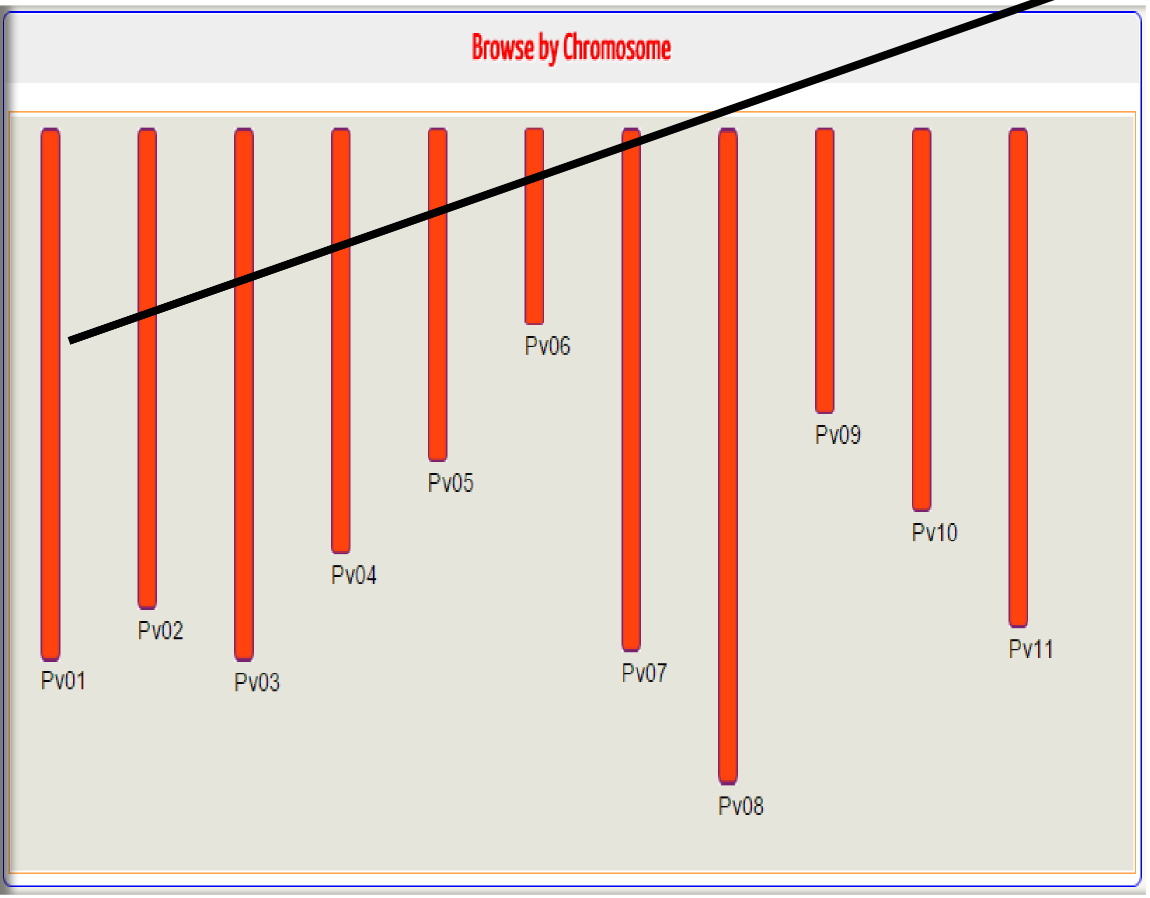

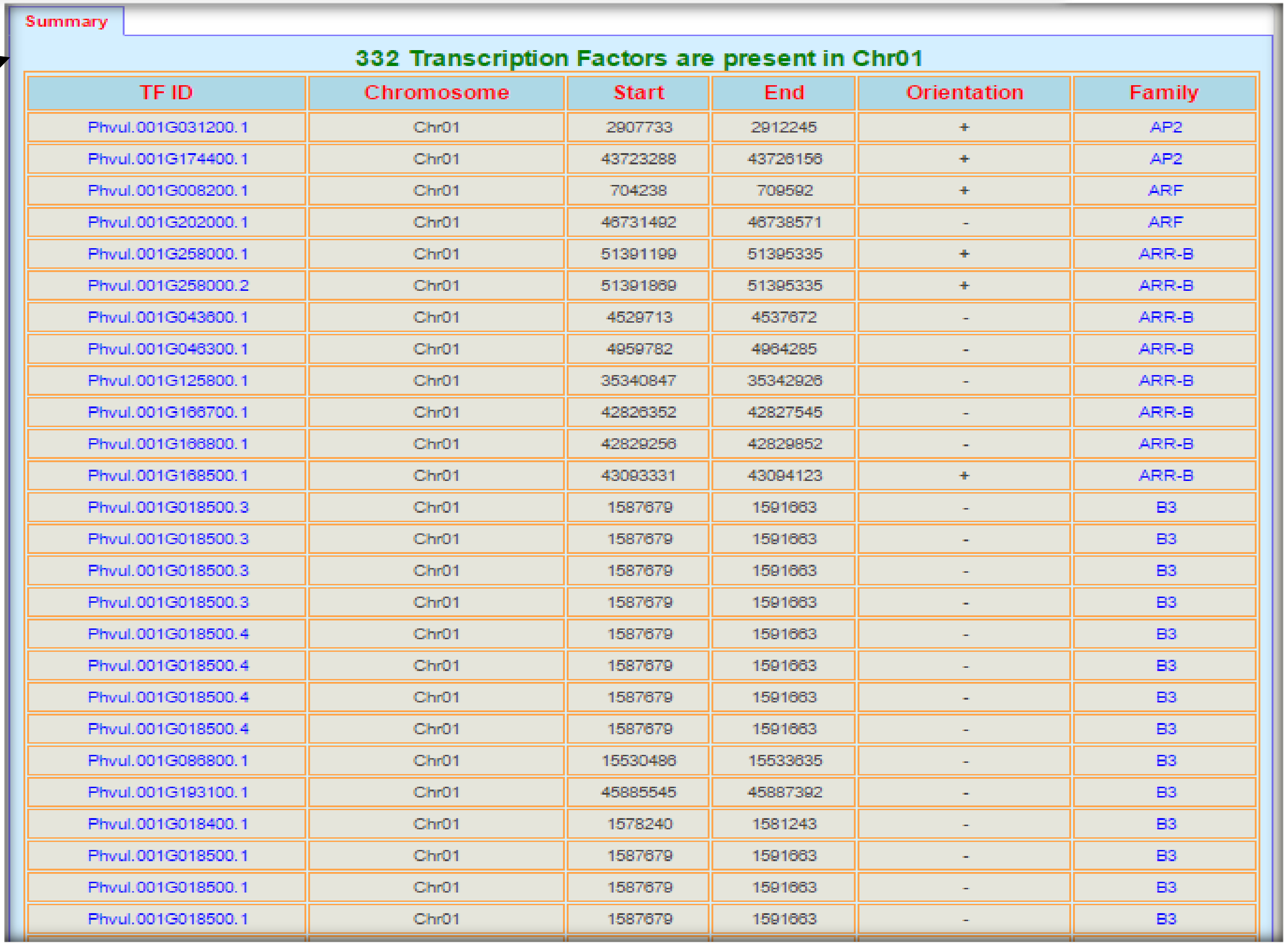

Summary

332 Transcription Factors are present in Chr01

TF ID	Chromosome	Start	End	Orientation	Family
Phvul.001G031200.1	Chr01	2907733	2912245	+	AP2
Phvul.001G174400.1	Chr01	43723288	43726156	+	AP2
Phvul.001G008200.1	Chr01	704238	709592	+	ARF
Phvul.001G202000.1	Chr01	46731492	46738571	-	ARF
Phvul.001G258000.1	Chr01	51391199	51395335	+	ARR-B
Phvul.001G258000.2	Chr01	51391869	51395335	+	ARR-B
Phvul.001G043600.1	Chr01	4529713	4537672	-	ARR-B
Phvul.001G046300.1	Chr01	4959782	4964285	-	ARR-B
Phvul.001G125800.1	Chr01	35340847	35342926	-	ARR-B
Phvul.001G166700.1	Chr01	42826352	42827545	-	ARR-B
Phvul.001G166800.1	Chr01	42829256	42829852	-	ARR-B
Phvul.001G168500.1	Chr01	43093331	43094123	+	ARR-B
Phvul.001G018500.3	Chr01	1587679	1591663	-	B3
Phvul.001G018500.3	Chr01	1587679	1591663	-	B3
Phvul.001G018500.3	Chr01	1587679	1591663	-	B3
Phvul.001G018500.3	Chr01	1587679	1591663	-	B3
Phvul.001G018500.4	Chr01	1587679	1591663	-	B3
Phvul.001G018500.4	Chr01	1587679	1591663	-	B3
Phvul.001G018500.4	Chr01	1587679	1591663	-	B3
Phvul.001G018500.4	Chr01	1587679	1591663	-	B3
Phvul.001G086800.1	Chr01	15530486	15533635	-	B3
Phvul.001G193100.1	Chr01	45885545	45887392	-	B3
Phvul.001G018400.1	Chr01	1578240	1581243	-	B3
Phvul.001G018500.1	Chr01	1587679	1591663	-	B3
Phvul.001G018500.1	Chr01	1587679	1591663	-	B3
Phvul.001G018500.1	Chr01	1587679	1591663	-	B3

REFERENCES

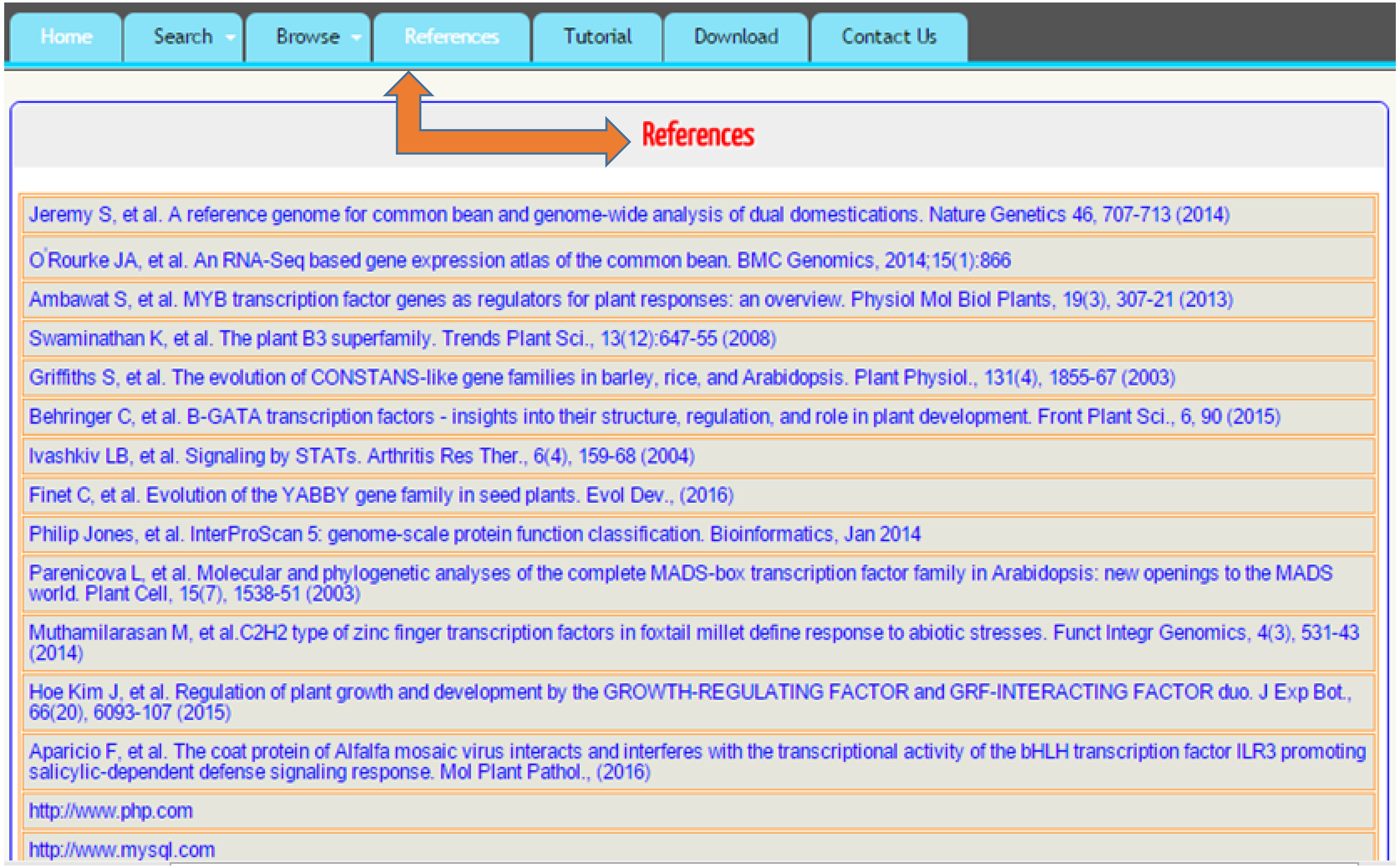
Home | Search | Browse | References | Tutorial | Download | Contact Us

References

Jeremy S, et al. A reference genome for common bean and genome-wide analysis of dual domestications. Nature Genetics 46, 707-713 (2014)

O'Rourke JA, et al. An RNA-Seq based gene expression atlas of the common bean. BMC Genomics, 2014;15(1):866

Ambawat S, et al. MYB transcription factor genes as regulators for plant responses: an overview. Physiol Mol Biol Plants, 19(3), 307-21 (2013)

Swaminathan K, et al. The plant B3 superfamily. Trends Plant Sci., 13(12):647-55 (2008)

Griffiths S, et al. The evolution of CONSTANS-like gene families in barley, rice, and Arabidopsis. Plant Physiol., 131(4), 1855-67 (2003)

Behringer C, et al. B-GATA transcription factors - insights into their structure, regulation, and role in plant development. Front Plant Sci., 6, 90 (2015)

Ivashkiv LB, et al. Signaling by STATs. Arthritis Res Ther., 6(4), 159-68 (2004)

Finet C, et al. Evolution of the YABBY gene family in seed plants. Evol Dev., (2016)

Philip Jones, et al. InterProScan 5: genome-scale protein function classification. Bioinformatics, Jan 2014

Parenicova L, et al. Molecular and phylogenetic analyses of the complete MADS-box transcription factor family in Arabidopsis: new openings to the MADS world. Plant Cell, 15(7), 1538-51 (2003)

Muthamilarasan M, et al.C2H2 type of zinc finger transcription factors in foxtail millet define response to abiotic stresses. Funct Integr Genomics, 4(3), 531-43 (2014)

Hoe Kim J, et al. Regulation of plant growth and development by the GROWTH-REGULATING FACTOR and GRF-INTERACTING FACTOR duo. J Exp Bot., 66(20), 6093-107 (2015)

Aparicio F, et al. The coat protein of Alfalfa mosaic virus interacts and interferes with the transcriptional activity of the bHLH transcription factor ILR3 promoting salicylic-dependent defense signaling response. Mol Plant Pathol., (2016)

http://www.php.com

http://www.mysql.com

DOWNLOAD

Home | Search | Browse | References | Tutorial | Download | Contact Us

Download Data

	Protein Sequences	CDS Sequences	Transcript Sequences	2kb Upstream Sequences	Genomic Sequences	SSRs
All TF Families	Download	Download	Download	Download	Download	Download

Please right click on "Download" and select "Save As" option to download the data conveniently.

CONTACT US

Home | Search | Browse | References | Tutorial | Download | Contact Us

Contact Us

Dr. MNV Prasad Gajula
MCA, MS(Sweden), PhD(Germany)
Research Scientist (Bioinformatics)& DST Ramanujan Fellow
Institute of Biotechnology,
PJTSAU, Rajendra Nagar,
Hyderabad-500030 India
Phone No: (0091) 96769-52555
Mail ID: gajula.ibt@gmail.com, varaprasad22@yahoo.com
My Profile @ SERB
